# Supplementary material for: Memantine and Riluzole Exacerbate, Rather Than Ameliorate Behavioral Deficits Induced by 8-OH-DPAT Sensitization in a Spatial Task
Source: Biomolecules. 2021 Jul 9;11(7):1007. doi: 10.3390/biom11071007 (PMC8301967; doi:10.3390/biom11071007)
Supplement: Supplementary file 1 [file biomolecules-11-01007-s001.zip › Analysis of intervals.pdf]

Analysis of intervals

We further compared the behavior of all groups during 5 consequent 10min intervals of the first and final sessions.

Locomotion

The analysis showed main effect of interval [F(4, 804.9) = 17.951, p < .001] and interval*session interaction F(4, 804.9) = 6.906, p < .001], but not group*interval interaction [F(24, 804.9) = 1.028, p = 0.427] and group*session*interval interaction [F(24, 804.9) = 0.760, p = 0.789], suggesting similar locomotor changes during the first and last session in all groups. As can be seen from Fig. S1A, locomotion decreased from the first to last interval in the first session in all groups. Although locomotion slightly decreased in the "saline" groups and slightly increased in the MEM-OH and the RIL-OH groups during the last session, the changes were very subtle.

Entrances

There was also significant main effect of interval [F(4, 805.2) = 16.226, p < .001] and interval*session interaction F(4, 805.2) = 12.224, p < .001], but not of group*interval interaction [F(24, 805.2) = 1.485, p = 0.063], suggesting number of entrances in all groups decreased from the beginning to the end of the first session (although in the "saline" groups more steeply) and remained stable in all groups during the last session (Fig. S1B). Also group*session*interval interaction was not significant [F(24, 805.2) = 0.543, p = 0.964].

Maximum time avoided

We found a significant effect of interval [F(4, 810) = 6.892, p < .001], interval*session [F(4, 810) = 10.74, p < .001] and group*interval interaction [F(24, 810) = 2.149, p = 0.001]. As can be seen from Fig. S1C, maximum time avoided increased in the "saline" groups, but not very much in the "OH" groups during the first session and it did not change very much in any group during the last session. Group*session*interval interaction was not significant [F(24, 810) = 0.572, p = 0.951].

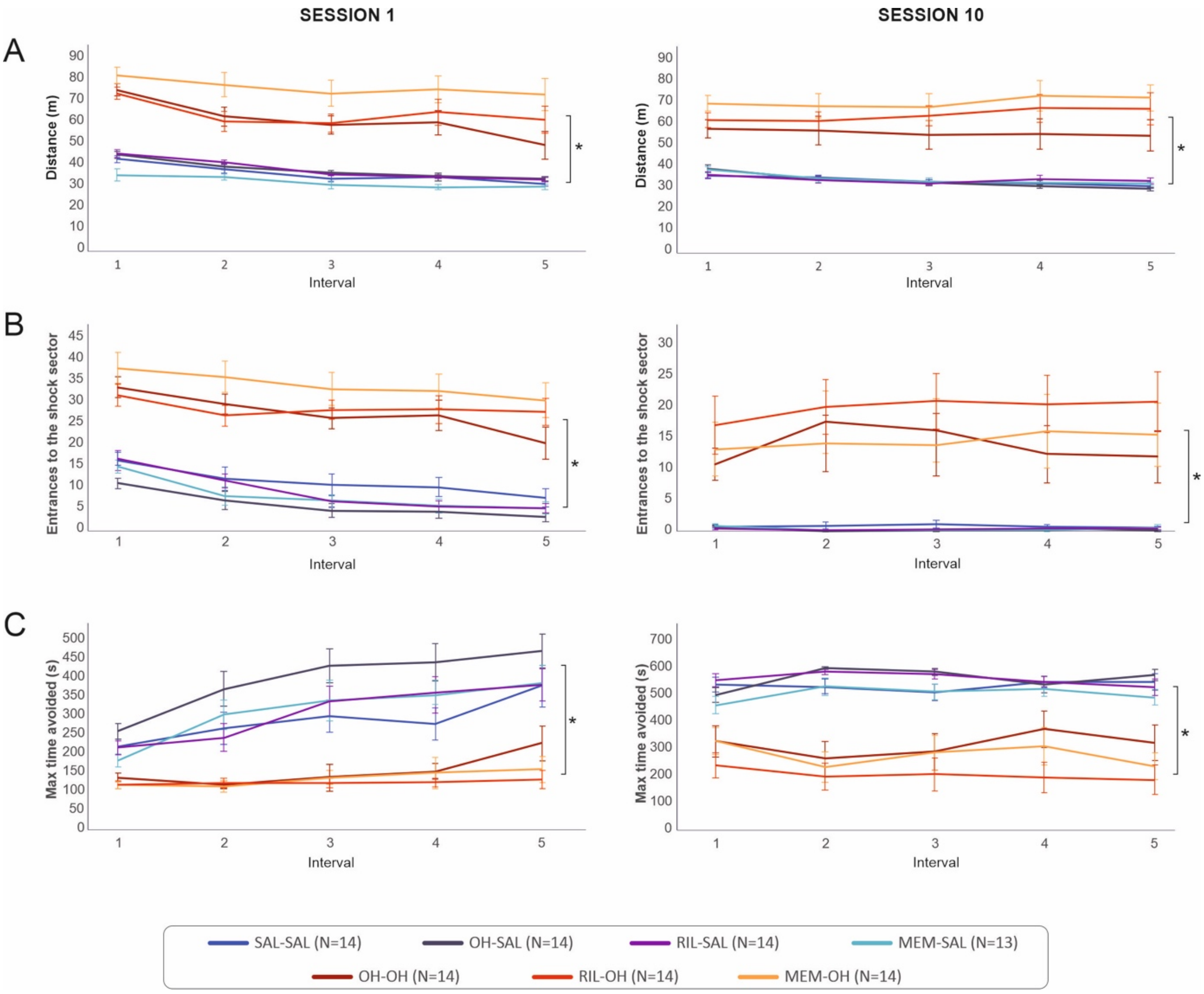

Figure S1: Analysis of intervals in the first and last acquisition sessions. (A) Locomotion of all treatment groups dissected into five consecutive 10-minute intervals. (B) Number of entrances. (C) Maximum time avoided of the shock sector. * denotes a significant difference at $p = 0.001$. Data are presented as mean values ± SEM
