# Supplementary material for: Memantine and Riluzole Exacerbate, Rather Than Ameliorate Behavioral Deficits Induced by 8-OH-DPAT Sensitization in a Spatial Task
Source: Biomolecules. 2021 Jul 9;11(7):1007. doi: 10.3390/biom11071007 (PMC8301967; doi:10.3390/biom11071007)
Supplement: Supplementary file 1 [file biomolecules-11-01007-s001.zip › Table of significant interactions.pdf]

Table S1. Significant interactions

	Distance			Entrances		
**session * interval**	***t***	***p***	**CI**	***t***	***p***	**CI**
1-10 * 0-10 x 10-20	2.088	0.037	[0.260, 8.193]	-2.895	0.004	[-1.051, -0.202]
**session x group**						
1-2 * RIL-OH x SAL SAL				-3.269	0.001	[-4.882, -1.223]
1-2 * RIL-OH x MEM-SAL				-2.984	0.003	[-4.615, -0.956]
2-3 * MEM-OH x OH-OH				2.355	0.019	[0.369, 4.027]
9-10 * MEM-OH x OH-OH				2.253	0.025	[0.276, 3.974]
	**Max time avoided**			**Median speed**		
**session * interval**	***t***	***p***	**CI**	***t***	***p***	**CI**
1-10 * 0-10 x 10-20	-2.328	0.020	[-3.188, -0.274]	-2.088	0.037	[-8.193, -0.260]
1-10 * 30-40 x 40-50	-2.463	0.014	[-3.288, -0.374]			
**session * group**						
1-2 * MEM-OH x SAL-SAL	2.393	0.017	[1.544, 15.537]	-2.464	0.014	[-5.529, -0.629]
1-2 * MEM-OH x OH-SAL				-3.276	0.001	[-6.544, -1.645]
1-2 * MEM-OH x RIL-SAL				-3.297	0.001	[-6.570, -1.671]
1-2 * MEM-SAL x MEM-OH				-2.815	0.005	[-6.081, -1.089]
1-2 * MEM-SAL x RIL-OH	2.851	0.004	[3.242, 17.502]			
1-2 * MEM-SAL x OH-OH	-2.428	0.015	[-15.963, -1.703]	-2.089	0.037	[-5.156, -0.164]
1-2 * OH-SAL x OH-OH	-2.181	0.030	[-14.781, -0.787]	-2.536	0.011	[-5.619, -0.720]

1-2 * RIL-SAL x OH-OH	-3.398	<.001	[-19.127, 5.134]	-2.557	0.011	[-5.645, -0.746]
1-2 * RIL-OH x SAL-SAL	2.325	0.020	[1.304, 15.297]			
2-3 * OH-SAL x SAL-SAL				-1.962	0.050	[-4.952, -0.003]
3-4 * OH-SAL x MEM-OH				-2.168	0.03	[-5.266, -0.265]
4-5 * RIL-SAL x OH-SAL				-2.091	0.037	[0.169, 5.229]
4-5 * RIL-SAL x SAL-SAL				3.210	0.001	[1.562, 6.462]
4-5 * RIL-SAL x MEM-OH				2.393	0.017	[0.541, 5.440]
4-5 * RIL-SAL x MEM-SAL				-2.2631	0.024	[-5.652, -0.406]
7-8 * RIL-SAL x SAL-SAL				-2.142	0.033	[-5.5494, -0.246]
8-9 * OH-OH x SAL-SAL	-2.362	0.018	[-15.429, -1.436]			
8-9 * OH-SAL x OH-OH	2.299	0.022	[1.211, 15.204]			
9-10 * MEM-OH x OH-OH	-1.956	0.051	[-14.125, 0.0160]			
9-10 * RIL-OH x OH-OH	-1.972	0.049	[-14.035, -0.042]			
9-10 * OH-SAL x OH-OH	-2.274	0.023	[-15.115, -1.122]			
**group * interval**	***t***	***p***	**95% CI**	***t***	***p***	**95% CI**
1-2 * OH-OH x OH-SAL	2.128	0.034	[0.232, 5.655]			
1-2 * OH-OH x MEM-SAL	-3.889	<.001	[-8.245, -2.719]			
1-2 * OH-OH x RIL-SAL	-2.254	0.024	[-5.829, -0.406]			
1-2 * OH-OH x OH-SAL	-3.678	<.001	[-7.799, -2.376]			
1-2 * OH-SAL x MEM-OH	3.410	<.001	[2.006, 7.429]			
1-2 * OH-SAL x RIL-OH	2.558	0.011	[0.828 6.251]			
1-2 * RIL-OH x MEM-SAL	2.790	0.005	[1.170, 6.696]			
