# Supplementary figures and images for: Memantine and Riluzole Exacerbate, Rather Than Ameliorate Behavioral Deficits Induced by 8-OH-DPAT Sensitization in a Spatial Task
Source: Biomolecules. 2021 Jul 9;11(7):1007. doi: 10.3390/biom11071007 (PMC8301967; doi:10.3390/biom11071007)

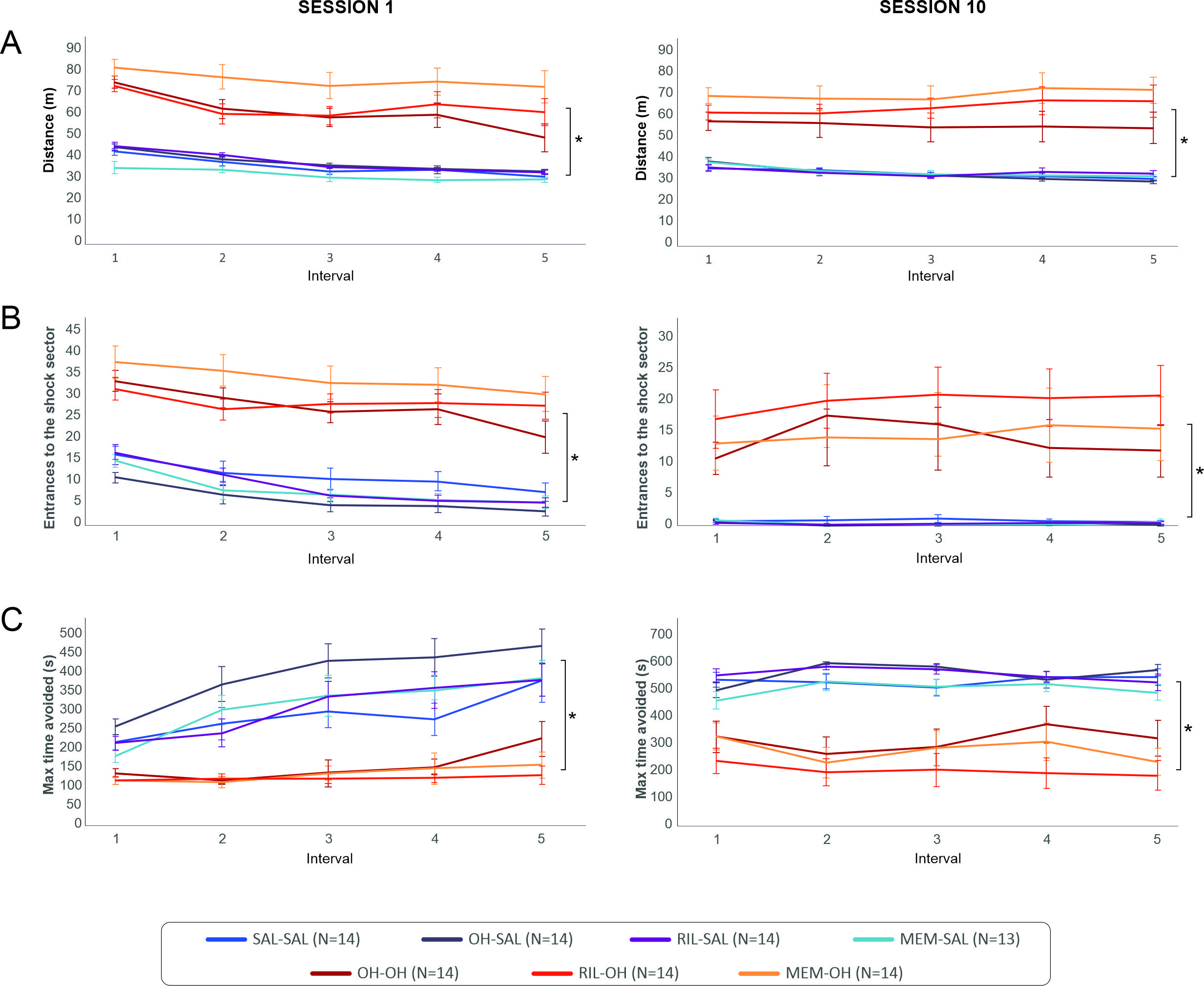

Supplement: Supplementary file 1 [file biomolecules-11-01007-s001.zip › figS1.jpg]
